# Downregulation of circular RNA circ-HN1 suppressed the progression of gastric cancer through the miR-485-5p/GSK3A pathway

**DOI:** 10.1080/21655979.2021.1987124

**Published:** 2022-02-21

**Authors:** Mingming Zhang, Yingheng Jiang

**Affiliations:** a Department of Gastrointestinal Surgery, Liuzhou People’s Hospital, Liuzhou, China; b Surgery Medical Insurance Office, Liuzhou People’s Hospital, Liuzhou, China

**Keywords:** Gastric cancer, circ-HN1, proliferation, migration, invasion

## Abstract

Gastric cancer (GC) is a malignancy with high incidence and mortality globally. Circular RNAs (circRNAs) are reported to regulate cellular processes in human diseases, including GC. Herein, the functions of circ-HN1 and its molecular mechanisms were investigated. circ-HN1, miR-485-5p, and GSK3A levels in GC were measured using Real time-quantitative polymerase chain reaction (RT-qPCR). Cell proliferation was analyzed using cell counting kit-8 (CCK-8) and colony formation assays. Meanwhile, the migration and invasion abilities were analyzed using the transwell assay. The targeted relationship was confirmed using a luciferase reporter assay and an RNA pull-down assay. In both GC tissues and cells, circ-HN1 expression was upregulated, and its silencing suppressed cellular processes. Moreover, circ-HN1 served as a sponge of miR-485-5p, which was reduced in patients with GC and negatively regulated by circ-HN1 in GC cells. Inhibition of miR-485-4p abolished the biological functions induced by the silencing of circ-HN1. Additionally, miR-485-5p targeted GSK3A in GC, whose expression was elevated in tumor tissues and was negatively correlated with miR-485-5p in tumor cells. GSK3A rescued the inhibition of miR-485-5p in the cellular processes. In conclusion, silencing of the circ-HN1–miR-485-5p–GSK3A regulatory network inhibited GC cell proliferation, migration, and invasion, suggesting that circ-HN1 is a potential target for GC therapy.

## Introduction

Gastric cancer (GC), the fifth most common cancer and the third leading cause of cancer death globally, is a heterogeneous disease that seriously influences human health and life [1]. The main risk factors for GC include old age, male sex, high salt intake, smoking, alcohol consumption, radiation, and family history [2,3]. Although the incidence of GC is declining and 5-year survival rates are improving, GC can be fatal in numerous patients because of delayed diagnosis at an advanced stage leading to the optimal time for surgery to be missed [3]. Furthermore, distant metastasis of tumor cells to other organs or tissues is also a major cause of cancer death or poor prognosis [4]. With the development of molecular-targeted therapy, combination with neoadjuvant chemoradiotherapy, and immunotherapy, the therapeutic outcomes of GC have significantly improved [5]. Therefore, the identification of novel targets for GC prevention and treatment contributes to the reduction of the incidence and mortality of GC.

Circular RNAs (circRNAs), which have closed loop structures but without 5ʹ to 3ʹ polarity or a polyadenylated tail, are an important type of non-coding RNAs [6]. circRNAs play a pivotal role in cellular homeostasis, development, and disease progression [7]. circRNA dysregulation is usually found in many diseases, including GC. An increasing number of circRNAs in GC have been identified to be involved in cellular functions such as proliferation, metastasis, and apoptosis [8]. Moreover, circRNAs play functional roles in GC by acting as sponges of microRNAs (miRNAs) and further regulating mRNA expression, which is called the circRNA–miRNA–mRNA regulatory network [9,10]. Circ-HN1 was newly discovered in GC using a microarray dataset; it is also known as circHN1_005 or circ_0045602, is 260 bp in length, and is produced by the reverse splicing of a linear transcript of exons 3–5 of the HN1 gene [11]. Previous studies have reported that circ-HN1 promotes GC development [11,12]. However, the functions of circ-HN1 and its potential mechanism of action have not been thoroughly studied.

The aim of this study was to explore the effect of circ-HN1 on the progression of GC. circ-HN1 serves as a tumor promoter in GC. Furthermore, the loss of circ-HN1 suppressed GC cellular processes by sponging miR-485-5p through the targeting of GSK3A. These data reveal a new regulatory network that facilitates GC progression, and suggest that circ-HN1 is potential for GC therapy.

## Materials and methods

### Tissue samples and cells

This study included 30 paired GC tissues and matched para-carcinoma tissues, which were obtained by surgery from patients with GC in Liuzhou People’s Hospital. After surgical excision, fresh tissues were stored at −80°C. None of the patients in the study received chemotherapy or radiotherapy. All patients provided written informed consent before the study. Our study was approved by the Ethics Committee of the Liuzhou People’s Hospital.

For *the in vitro* study, human gastric mucosal epithelial GES-1 and GC cell lines, including HGC-27, AGS, SGC-7901, and MKN-28, were acquired from ATCC (Manassas, VA, USA). All cells were cultured in RPMI-1640 (Gibco, Grand Island, NY, USA) supplemented with 10% fetal bovine serum (FBS; Beyotime, Shanghai, China) and 1% penicillin/streptomycin (Beyotime) and were incubated in a humidified incubator at 37°C with 5% CO_2_.

### Cell transfection

Transfection of small interfering RNAs (siRNAs: si-nc, si-circ-HN1) and plasmids (overexpression vector, circ-HN1, Ad-NC, and Ad-GSK3A) was performed using Cobioer (Nanjing, China). Mimics (miR-485-5p mimic and NC) and inhibitors (miR-485-5p inhibitor and NC) were acquired from GenePharma (Shanghai, China). Lipofectamine 2000 (Invitrogen, Carlsbad, CA, USA) was used for transient transfection. Reverse-transcription quantitative polymerase chain reaction (RT-qPCR) was performed 48 h post-transfection.

### Detection of the proliferation ability

Cell proliferation was assessed using the cell counting Kit-8 (CCK-8) assay (MadChemExpress, Shanghai, China). Transfected cells were placed in 96-well plates, which were maintained in an incubator for 0, 24, 48, and 72 h. After adding10 μl of CCK-8 solution to the plates, the cells were maintained in the incubator for 1 h. The optical density (OD) value at 450 nm was read using a microplate reader (Molecular Devices, Shanghai, China) after mixing gently on an orbital shaker for 1 min.

Cell proliferation was analyzed using a colony formation assay. Cells were incubated for 2 weeks after transfection. After fixing with 4% paraformaldehyde, the cells were stained with 0.1% crystal violet at room temperature. The number of colonies was counted using an inverted microscope (Olympus, Tokyo, Japan).

### Detection of the migration and invasion ability

Twenty-four-well transwell chambers with an 8-µm pore size were obtained from Corning Incorporated (Corning, NY, USA). The upper chambers coated with Matrigel (BD Biosciences, San Jose, CA, USA) were used for the invasion assay, and the chambers without Matrigel were used for the migration assay. Cells post-transfection were resuspended in RPMI-1640 without serum and were plated in the upper chambers, while complete RPMI-1640 (600 μl) was added to the lower chambers. After 24 h, the cells were removed from the upper chamber. Other cells that migrated or invaded the lower surface were fixed with 4% paraformaldehyde and stained with 0.1% crystal violet. Five random fields in each chamber were imaged using an inverted microscope (Olympus). The numbers were quantified.

### RT-qPCR

All kits used in this experiment were purchased from Tiangen (Beijing, China), and each process step was performed in accordance with the manufacturer’s instructions. Total RNA and miRNA were isolated from tissues and cultured cells using the RNAsimple Total RNA Kit and miRcute miRNA isolation kit, respectively. RT and qPCR were performed using the FastKing One Step RT-qPCR Kit (SYBR Green) on a Real Time System (BioRad, Hercules, CA, USA). The conditions of RT and qPCR were as follows: 50°C for 30 min (RT), 95°C for 3 min (pre-degeneration), 40 cycles of 95°C for 15 s (degeneration), and 60°C for 30 s (annealing and elongation). circ-HN1 and GSK3A expression was normalized to GAPDH expression, and miR-485-5p expression was normalized to U6 snRNA expression. The 2^–ΔΔCT^ method was used to calculate the relative expression. The specific primers sequences (5ʹ–3ʹ) are listed as follows: circ-HN1 F: GCAGGGAAGACTTGGAGTCA; R: AAAATTGGATCCACCACCTG, GSK3A F: GGAAAGGCATCTGTCGGGG; R: GAGTGGCTACGACTGTGGTC, and miR-485-5p F: TGCGCTCAGCAAACATTTATTG; R: CCAGTGCAGGGTCCGAGGT.

### Rnase R treatment

Total RNA (10 µg) was incubated with or without 40 U Rnase R (BioVision, Milpitas, CA, USA) at 37°C for 30 min. The expression of HN1 and circ-HN1 was measured using Rt-qPCR.

### Bioinformatic analysis

The potential interaction between circ-NH1 and miR-485-5p was predicted using Circular RNA Interactome online tool (https://circinteractome.irp.nia.nih.gov/index.html). The interaction between miR-485-5p and GSK3A was predicted using TargetScanHuman 7.2 online tool (http://www.targetscan.org/vert_72/).

### Luciferase reporter assay

GC-7901 and MKN-28 cells were placed in 24-well plates in the incubator. The wild-type (WT) or mutant (MUT) 3-UTR of circ-HN1 and the WT or MUT 3′-UTR of GSK3A were cloned into pGL3 vectors (Promega, Madison, WI, USA) to construct luciferase reporter plasmids. Lipofectamine 2000 (Invitrogen) was co-transfected with WT or MUT plasmids and mimic plasmids into cells. A dual luciferase reporter assay system (Promega) was used to assess the relative luciferase activity after 48 h of transfection. *Renilla* luciferase activity was used as an endogenous reference for firefly luciferase activity.

### Biotinylated miRNA pull-down assay

The transfected cells were labeled with biotin-miRNA (miR-485-5p) for 72 h. The cells were then incubated with a lysis buffer. Lysates were used as the input group. Dynabeads® M-280 Streptavidin (Invitrogen) was mixed with remnant lysates for 3 h at 4°C. Then, the harvested mixture was added to the lysate using lysis buffer. After RNA complex extraction using the RNeasy Mini Kit (Qiagen, Duesseldorf, Germany), the enrichment of circ-HN1 and GSK3A was measured by RT-qPCR.

### Statistical analysis

All data from at least three independent experiments are shown as mean ± SD. Student’s *t*-test and one-way ANOVA were used to analyze the differences using GraphPad Prism (Version 6; La Jolla, CA, USA). Statistical significance was defined as P < 0.05.

### Results

The study explored the role of circ-HN1 in GC and investigate the potential molecular mechanism. We studied the effect of circ-HN1 knockdown on cell proliferation, migration, and invasion. We found that knockdown of circ-HN1 suppressed the proliferation, migration, and invasion of GC cells via the miR-485-5p/GSK3A axis. The study will provide a new target for GC therapy.

#### Circ-HN1 was upregulated in GC

**Figure 1.**
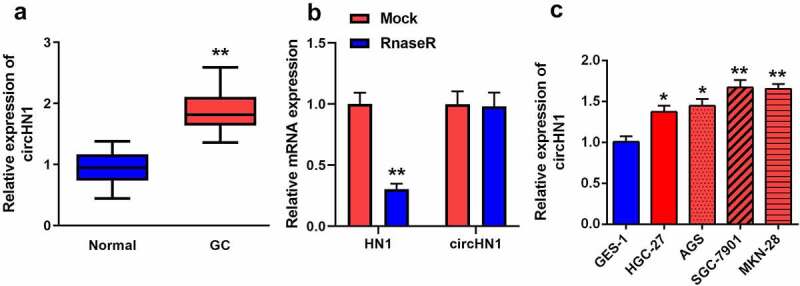
Circ-HN1 expression upregulated in GC. (a) RT-qPCR was conducted to test the relative expression of circ-HN1 in tumor tissues and matched para-carcinoma tissues. (b) Relative RNA expression was detected using RT-qPCR after Rnase R treatment. (c) Circ-HN1 expression evaluated by RT-qPCR in gastric mucosal epithelial GES-1 cells and GC cells (HGC-27, AGS, SGC-7901 and MKN-28). **P < 0.01. *P < 0.05.

Circ-HN1 levels in GC were measured by RT-qPCR. circ-HN1 levels were elevated in GC tissues than in tumor-adjacent tissues (Figure 1a). Compared with HN1, circ-HN1 was significantly resistant to Rnase R (Figure 1b). Similarly, in GC cell lines, circ-HN1 levels were increased in HGC-27, AGS, SGC-7901, and MKN-28 cells compared to that in GES-1 cells (Figure 1c). Both GC-7901 and MKN-28 cells were used for the subsequent experiments.

#### Decreased expression of circ-HN1 restrained GC cellular processes

**Figure 2.**
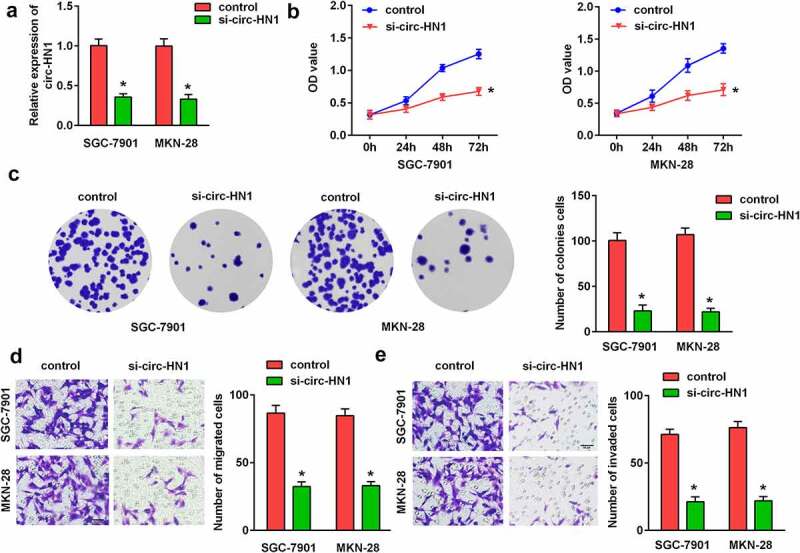
Silencing of circ-HN1 suppressed GC cell progression. (a) Expression of circ-HN1 in the control (si-nc) and si-circ-HN1 groups was examined by RT-qPCR. (b) Cell proliferation was assessed using the CCK-8 assay and the optical density was measured at 490 nm. (c) Cell proliferation was assessed using the colony formation assay. (d) Migrated cells were quantified using the Matrigel-free transwell assay. (e) Cell invasion ability was analyzed using the Matrigel transwell assay. **P < 0.01. *P < 0.05.

To explore the physiological impact of circ-HN1, si-nc and si-circ-HN1 were transfected into GC-7901 and MKN-28 cells. As illustrated in Figure 2a, circ-HN1 expression was reduced by circ-HN1 loss, suggesting that the cells were successfully transfected. The CCK-8 assay and colony formation assay revealed that the silencing of circ-HN1 restrained the proliferation of GC cells (Figure 2b and c). According to the transwell assay, the migration and invasion abilities were inhibited by circ-HN1 knockdown (Figure 2d-e).

#### Circ-HN1 sponged miR-485-5p in GC

**Figure 3.**
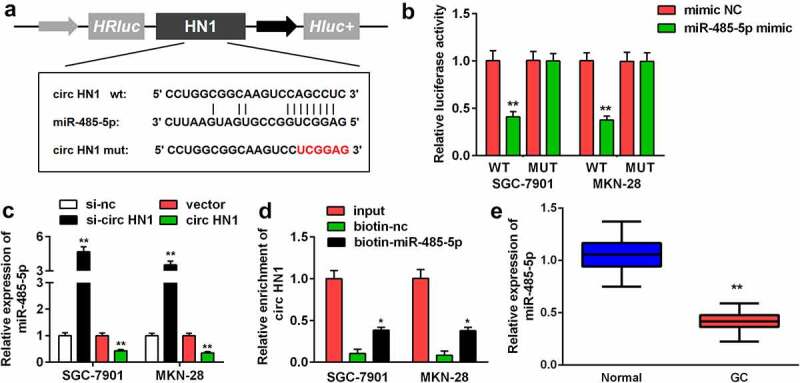
Circ-HN1 sponged miR-485-5p. (a) Potential of the combination of miR-485-5p and the designed circ-HN1 MUT. (b) GC-7901 and MKN-28 cells were co-transfected mimic plasmids and circ-HN1 MUT/WT, and the luciferase activity was evaluated. (c) Circ-HN1 expression was detected by RT-qPCR in GC cells transfected with si-circ-HN1 and circ-HN1. (d) The RNA pull-down assay was conducted using RT-qPCR with input, biotin-nc and biotin-miR-485-5p. (e) MiR-485-5p expression was evaluated in GC tissues and para-carcinoma tissues using RT-qPCR. **P < 0.01. *P < 0.05.

To explore the molecular mechanism of the action of circ-HN1, circ-HN1 WT was noted to have the potential to bind to miR-485-5p. A circ-HN1 MUT sequence was designed (Figure 3a). MiR-485-5p reduced the relative luciferase activity of circ-HN1 WT in both GC-7901 and MKN-28 cells (Figure 3b). MiR-485-5p expression was upregulated with si-circ-HN1, which was decreased by circ-HN1 (Figure 3c). Additionally, circ-HN1 was enriched with biotin-miR-485-5p (Figure 3d). Compared to that in paracancerous tissues, miR-485-5p was downregulated in GC tissues (Figure 3e).

#### Inhibition of miR-485-5p reversed the circ-HN1-induced effects on biological functions

**Figure 4.**
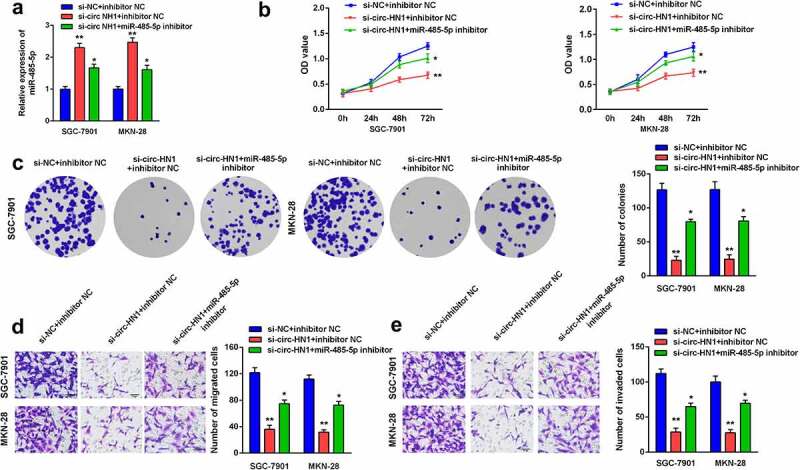
Downregulation of miR-485-5p rescued the circ-HN1 silencing-induced effects on biological functions. (a) MiR-485-5p levels after transfection were measured using RT-qPCR. (b) Cell proliferation after transfection was evaluated using the CCK-8 assay. (c) Cell proliferation was evaluated using the colony formation assay. (d) Cell migration and (e) Cell invasion abilities were evaluated using the transwell assay. **P < 0.01. *P < 0.05.

Furthermore, rescue assays were performed by transfection of si-circ-HN1 and miR-485-5p inhibitor. MiR-485-5p was upregulated by circ-HN1 knockdown, which was subsequently abrogated by miR-485-5p inhibition (Figure 4a). Cell proliferation, migration, and invasion were inhibited by si-circ-HN1, which were subsequently abolished by the downregulation of miR-485-5p (Figure 4b-e).

#### MiR-485-5p targeted GSK3A in GC

**Figure 5.**
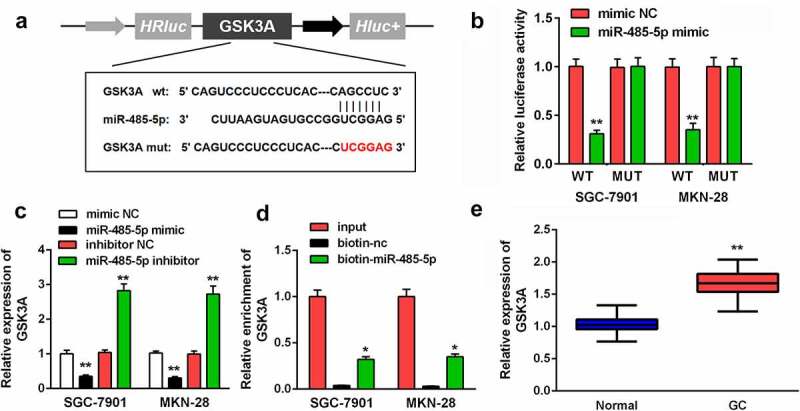
MiR-485-5p targeted GSK3A. (a) MiR-485-5p bound to the 3ʹ-UTR of GSK3A. GSK3A MUT was also designed. (b) The relative luciferase activity after co-transfection of mimic plasmids and WT/MUT plasmids was analyzed in GC cells. (c) GSK3A levels in GC cells after the transfection of miR-485-5p mimic or inhibitor were evaluated using RT-qPCR. (d) RNA pull-down assay was conducted with input, biotin-nc and biotin-miR-485-5p, and GSK3A expression was measured by RT-qPCR. (e) GSK3A expression in GC tissues and pericarcinomatous tissues was examined using RT-qPCR. **P < 0.01. *P < 0.05.

One of the targets of miR-485-5p was further investigated. The possibility of GSK3A combining with miR-485-5p is shown in Figure 5a. The relative luciferase activity was decreased by miR-485-5p in GSK3A WT-transfected cells, compared with mimic NC (Figure 5b). In addition, GSK3A expression was negatively correlated with miR-485-5p expression; GSK3A levels were upregulated on transfecting the miR-485-5p inhibitor and were downregulated on transfecting the miR-485-5p mimic (Figure 5c). GSK3A levels were enriched by biotin-miR-485-5p (Figure 5d). Moreover, GSK3A expression was higher in tumor tissues than in the corresponding non-tumor tissues (Figure 5e).

#### GSK3A reversed the effects of miR-485-5p on GC cell progression

**Figure 6.**
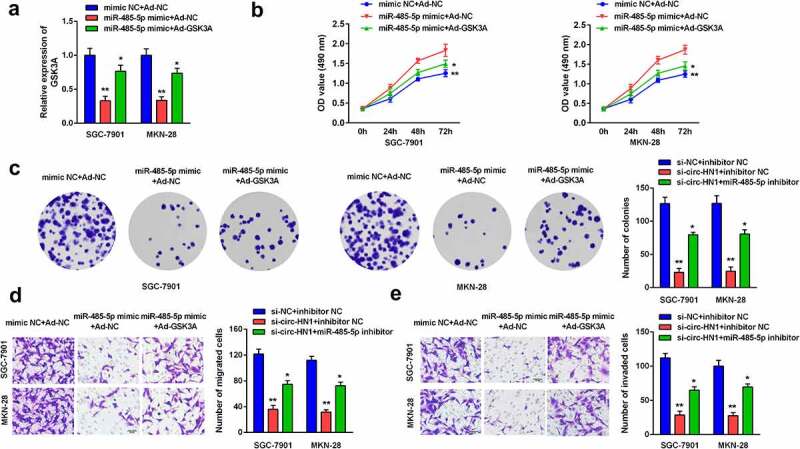
MiR-485-5p-induced effects on biological functions were rescued by GSK3A. (a) GSK3A expression in cells overexpressing miR-485-5p and GSK3A was measured using RT-qPCR. (b) Cell proliferation was assessed using the CCK-8 assay. (c) Cell proliferation was also assessed using the colony formation assay. (d) Cell migration and (e) cell invasion abilities was analyzed using the transwell assay. **P < 0.01. *P < 0.05.

Post-transfection, GSK3A expression was reduced by miR-485-5p overexpression, which was further reversed by Ad-GSK3A (Figure 6a). Overexpression of miR-485-5p repressed cell proliferation, while overexpression of GSK3A abolished this suppression (Figure 6b and c). Furthermore, cell migration and invasion were inhibited by miR-485-5p, which were abrogated by GSK3A, as analyzed by the transwell assay (Figure 6d and e).

## Discussion

Many studies have reported the involvement of circRNAs in the development of human cancers, including GC. Aberrant expression of circRNAs in cancers commonly serves as a tumor suppressor or tumor promoter. For example, the loss of circ_001988 accelerates cell proliferation, invasion, and migration, whereas the overexpression of circ_001988 inhibits heterograft tumor growth in GC [13]. Additionally, downregulated circ_0004872 expression has been identified in GC tissues, which suppresses GC cell proliferation, invasion, and migration [14]. In contrast, circ-RanGAP1 silencing attenuates GC cell invasion and migration, and represses tumor growth and metastasis in mice [15]. Ding et al. [16] have revealed that circ-DONSON is upregulated in tumor tissues and its knockdown restrains cell proliferation, migration, and invasion in GC. The expression of the HN1 gene is commonly elevated in tumors and contributes to accelerated tumor progression in breast cancer, anaplastic thyroid carcinoma, prostate cancer, and hepatocellular carcinoma [17-20]. CircHN1, encoded by the HN1 gene, can promote proliferation, migration, and invasion, as well as inhibit apoptosis in GC [11,12]. In the current study, circ-HN1 expression was found to be increased in GC rather than linear HN1, and knockdown of circ-HN1 attenuated GC cell biological progression. These results illustrated that circ-HN1 has tumor-promoting functions in GC, which is consistent with the findings of previous research.

Generally, circRNAs are stable molecules that regulate gene expression by acting as efficient sponges for miRNAs [10]. Wang et al. [11] reported that miR-302b-3p is a downstream target of circHN1. In this study, we confirmed that circ-HN1 could sponge the downstream molecule miR-485-5p using a luciferase reporter assay and a biotinylated miRNA pull-down assay. Moreover, circ-HN1 and miR-485-5p levels in GC were negatively correlated, which verified their targeted relationship. The effects of miR-485-5p have been explored in human diseases, including osteoporosis, cardiac hypertrophy, and malignancies [21-23]. In human cancers, miR-485-5p commonly plays a tumor-suppressive role. Chen et al. [22] revealed that miR-485-5p represses cell metastasis and the EMT process in bladder cancer. In addition, the migration and invasion of breast cancer cells is inhibited by the overexpression of miR-485-5p [24]. Additionally, it slows down the progression of acute myeloid leukemia, esophageal cancer, and osteosarcoma [25-27]. Similarly, the inhibitory functions of miR-485-5p were also found in GC. miR-485-5p levels are decreased in GC tissues, and its downregulation accelerates cell proliferation, migration, invasion, and EMT [28,29]. Moreover, lower miR-485-5p levels indicate poor prognosis in patients with GC [30]. Our study demonstrated that miR-485-5p expression was decreased in GC tissues, which is consistent with the findings of previous research. Moreover, miR-485-5p reversed the suppression of cellular processes induced by circ-HN1 silencing. These findings suggest that the loss of circ-HN1 repressed the progression of GC by sponging miR-485-5p.

miRNAs modulate gene expression in diseases by promoting mRNA degradation and/or inhibiting mRNA translation [31]. We identified GSK3A as a target of miR-485-5p. GSK-3 is a serine-threonine kinase containing both GSK-3α and GSK-3β isozymes, which are encoded by GSK3a and GSK3b, respectively [32]. GSK3A is found as a member of the Wnt/β-catenin pathway; its activation is usually observed in malignancies and is associated with cell processes and tumor recurrence [33,34], indicating its involvement in the evolution of cancers. Previous studies have demonstrated the dysregulation or dysfunction of GSK3A in breast cancer, oral cancer, and acute myeloid leukemia [35-37]. However, to date, there have been no studies on the biological functions of GSK3A in GC. In the current study, GSK3A expression was found to be enhanced in tumor tissues. Moreover, GSK3A rescued the biological functions of GC cells that were inhibited by the action of miR-485-5p. These data suggest that miR-485-5p targets GSK3A to suppress the progression of GC. Taken together, circ-HN1 exerts inhibitory effects on cellular processes by sponging miR-485-5p and the subsequent targeting of GSK3A.

## Conclusions

Our study identified that circHN1 functions as a tumor-promoting regulator in GC; the silencing of circHN1 inhibited cell proliferation, migration, and invasion. Moreover, we found that circHN1 directly targeted miR-485-5p, which further targeted GSK3A. This circHN1–miR-485-5p–GSK3A regulatory network plays an important role in GC, and these findings provide a fundamental basis for the therapeutic application of circHN1 in GC.

## Research highlights

1. Circ-HN1 is upregulated in GC tissues and cells.

2. Circ-HN1 silencing suppressed GC cell proliferation, migration and invasion.

3. Circ-HN1 sponges miR-485-5p, and miR-485-5p targets GSK3A.

4. Silencing of circHN1–miR-485-5p–GSK3A axis alleviates the progression of GC.
